# Identification and Characterization of Spontaneous Auxotrophic Mutants in *Fusarium langsethiae*

**DOI:** 10.3390/microorganisms5020014

**Published:** 2017-03-31

**Authors:** Olga Gavrilova, Anna Skritnika, Tatiana Gagkaeva

**Affiliations:** All-Russian Institute of Plant Protection (VIZR), St.-Petersburg, Pushkin 196608, Russia; olgavrilova1@yandex.ru (O.G.); orina-alex@yandex.ru (A.S.)

**Keywords:** auxotrophy, subgroups of *F. langsethiae*, temperature sensitivity, biotin, thiamine, media, northern Europe

## Abstract

Analysis of 49 strains of *Fusarium langsethiae* originating from northern Europe (Russia, Finland, Sweden, UK, Norway, and Latvia) revealed the presence of spontaneous auxotrophic mutants that reflect natural intraspecific diversity. Our investigations detected that 49.0% of *F. langsethiae* strains were auxotrophic mutants for biotin, and 8.2% of the strains required thiamine as a growth factor. They failed to grow on vitamin-free media. For both prototrophic and auxotrophic strains, no growth defect was observed in rich organic media. Without essential vitamins, a significant reduction in the growth of the auxotrophic strains results in a decrease of the formation of T-2 toxin and diacetoxyscirpenol. In addition, all analysed *F. langsethiae* strains were distinguished into two subgroups based on PCR product sizes. According to our results, 26 and 23 strains of *F. langsethiae* belong to subgroups I and II respectively. We determined that the deletion in the intergenic spacer (IGS) region of the rDNA of *F. langsethiae* belonging to subgroup II is linked with temperature sensitivity and causes a decrease in strain growth at 30 °C. Four thiamine auxotrophic strains were found in subgroup I, while 21 biotin auxotrophic strains were detected in subgroups II. To the best of our knowledge, the spontaneous mutations in *F. langsethiae* observed in the present work have not been previously reported.

## 1. Introduction

In 2004, M. Torp and H. Nirenberg described *Fusarium langsethiae* as a new species [1]. Later, molecular methods used in combination with traditional morphological methods revealed that *F. langsethiae* resembles *F. poae*, *F. sporotrichioides* and *F. sibiricum* phylogenetically [2,3,4,5]. Previous phylogenetic studies have shown that *F. langsethiae* strains were possible to divide into two subgroups based on ribosomal intergenic spacer (IGS) sequences [3,4,5]. 

*F. langsethiae* has been isolated from small grain cereals (oats, wheat, barley and triticale) in Northern Europe, but is currently detected in nearly all territories in the north and south of Europe [6,7,8,9,10,11,12].

From the time information about *F. langsethiae* first came to the forefront, the interest in this particular species has increased dramatically. Based on investigations, *F. langsethiae* together with closely related *F. sporotrichioides* are the main type-A trichothecene (T-2/HT-2 toxins) producers [13]. Despite the considerable efforts made by researchers, the life cycle, ecology and transmission of *F. langsethiae* are not fully understood. The inoculation of plants by *F. langsethiae* is typically unsuccessful, where the symptomless infection on cereal crops indicates that *F. langsethiae* is either an endophyte or saprophyte and a weak pathogen [8,11,14,15].

Variability was noted in aggressiveness in in vitro detached leaves among *F.* *langsethiae* isolates, which were not dependent on the source from which they were isolated (oats or wheat) [8,16]. Significant differences in toxin-producing ability were not observed with regard to the origin of isolates or host plants [17,18,19].

Many abiotic factors can significantly affect sporulation, toxin production ability and other characteristics of *F. langsethiae* strains. Consequently, it is important to understand the environmental aspects that affect fungal growth.

A temperature of 25 °C has been previously reported to be optimal for *F. langsethiae* growth and T-2/HT-2 toxin production [20,21,22]. However, 15 °C was also shown to be optimal for toxin production [17].

Cultivation of two *F. langsethiae* strains isolated from durum wheat in southern Italy at eight temperatures revealed that the colony growth and sporulation of both strains were the highest between 20 and 25 °C [23,24]. The colony diameter increased between 5 and 20 °C and there was no growth at 35 or 40 °C. It showed that the temperature and interaction of strain and temperature were significant factors, whereas strain alone was not.

The nutrient medium is the major factor that influences cultivated fungi. The media determine the colony morphology and pigmentation, formation of particular structures and whether a fungus will even grow in culture [25]. All fungi require several specific elements for growth and reproduction. This requirement is particularly important for auxotrophic mutants that are unable to synthesize a particular organic compound needed for growth.

This paper reports the isolation and characterization of auxotrophic strains of *F. langsethiae* belonging to two IGS-subgroups among 49 strains originating from Northern Europe. We expect that our study will contribute to a better comprehension of the genetic diversity of *F. langsethiae*, which is essential for control strategies to minimize the mycotoxins content in grain.

## 2. Materials and Methods

### 2.1. Fungal Strains

A total of 49 *F. langsethiae* strains from northern Europe were examined (28 originating from the north-western part of Russia, 11 from Finland, 5 from Sweden, 3 from England, 1 from Latvia and 1 from Norway). Some strains were provided to us by our colleagues Drs. T. Yli-Mattila (Finland), S. Edwards (UK) and J. Fatehi (Sweden). Other strains were isolated from cereal grains by the authors. All *Fusarium* strains analysed in this study were single-spored and stored in the All-Russian Plant Protection Institute collection (VIZR, St. Petersburg, Pushkin, Russia). The geographic origin, host and year of the strain isolations are listed in Table 1. Some of the strains had been studied previously, and information pertaining to those particular strains was published, e.g., toxin production ability [5,26] and IGS sequences [5]. 

### 2.2. DNA Isolation and PCR Amplification

DNA was extracted from fungal cultures in accordance with the procedures of the Genomic DNA Purification Kit (Thermo Fisher Scientific) and the DNA samples were stored at −20 °C prior to analysis by qualitative PCR. The DNA quality of each strain was confirmed by using ITS1/ITS4 primers [27]. The species-specific primer pair PfusF/FlanR for *F. langsethiae* [4] was used in PCR reactions. The primers CNL12/PulvIGS [3] were used to distinguish two *F. langsethiae* IGS-subgroups based on the PCR product sizes. All primers were synthesized by Evrogen Co. (Russia, Moscow). The PCR procedures were carried out according to the protocols mentioned by the authors who designed the primers.

PCR amplifications were performed in a reaction volume of 20 μL in a thermal cycler C1000 (Bio-Rad, Hercules, CA, USA). Aliquots (10 μL) of each PCR product were analysed by electrophoresis in a Tris-borate-EDTA buffer in 1.0% agarose gels. The sizes of the PCR products were visualized using the ChemiDoc MP Imaging System (Bio-Rad, Hercules, CA, USA). 

### 2.3. Culture Media for Fungi Growth

In this study, we used handmade potato-sucrose agar (PSA) as the medium for the isolation and identification of *F. langsethiae*. The medium contained 15 g of agar, 15 g of sucrose and broth from 200 g of scrubbed and diced fresh white potatoes per litre of distilled water [28].

Spezieller nährstoffarmer agar (SNA) was used to maintain *Fusarium* cultures [29]. An agar disc with diameter 5.0 mm of *F. langsethiae* grown on SNA for 10 to 20 days was typically used for sub-culturing strains in agarized or liquid media.

Basal Czapek (CZ) medium lacking vitamins contains (in g/L): sucrose or dextrose—15, sodium nitrate—2.0; dipotassium phosphate—1.0; magnesium sulphate—0.5; potassium chloride—0.5; and ferrous sulphate—0.01. In the experiments, both agarized (15 g/L) and liquid nutrient forms of CZ media were used.

Sterilization of the required material (media, needles and scalpels) was performed by autoclaving at 121 °C for 30 min. The final pH of the media was 5.9–6.0.

### 2.4. Morphological Studies

The strains were grown on agarized media in darkness at 24 °C to observe the surface and reverse of the colony. The phenotype of the fungal strains was assessed by direct observation of the growth of the cultures on media in darkness for a fortnight. The microstructures of strains were observed using an AxioVision Viewer 4.8 microscope (Carl Zeiss, Jena, Germany). 

To estimate the growth rate, each strain was cultivated on agar media (PSA and CZ). Mycelial plugs of each strain obtained from the colony growth on SNA were individually placed surface downward on the media in the centre of each Petri dish. The mycelial growth rate per day was calculated based on the differences between the colony diameters (average of two perpendiculars transects per plate) on the 3rd and 7th days of growth in darkness. The effects of temperature on growth were examined by incubating cultures on agar media at 15, 24 and 30 °C. 

To visualize the differences in the growth of the 19 strains, their biomasses were compared by cultivating them on liquid CZ. Therefore, each strain was cultured in a 750-mL Erlenmeyer flask containing 100 mL of liquid medium and incubated at 24 °C in shaker moving at 50 rpm for 10 days. Each flask was inoculated with diameter 5.0 mm mycelial plugs obtained from the colonies growing on SNA. To determine the biomass, the culture supernatant was separated from the mycelium by passing it through a filter paper via a vacuum pump (Millipore XF5423050, Alsace, France). The biomass was dried at 50 °C until complete dryness was achieved and then weighed. Each set of conditions and experiments was replicated at least twice.

### 2.5. Mycotoxin Analysis

The T-2 toxin and diacetoxyscirpenol (DAS) production ability of each strain was evaluated after cultivation on 1 mL of agarized medium (PSA or CZ) in 10 mL glass flasks for 7 days at 24 °C. One millilitre of acetonitrile and water mixture (84:16, *v*/*v*) was added to the flask, and the mixture was intensively shaken for 14 to 16 h at room temperature (shaker ELMI, Rīga, Latvia). The mycotoxin content was determined in the extracts using qualified test systems (VNIIVSGE, Moscow, Russia) for enzyme-linked immunosorbent assay (indirect ELISA), with a detection sensitivity of 0.02 ppm.

### 2.6. Identification of Auxotrophic Mutants

Based on the growth, all strains of *F. langsethiae* on CZ were classified as prototrophs and auxotrophs because the latest strains were characterized by weak or no growth on the said media. 

*F. langsethiae* strains that failed to grow on CZ were screened using the modified cross-pool auxanography scheme propounded by Holliday [30] to identify their specific auxotrophic requirements. The basal CZ medium used in the nutritional studies was modified by supplementation with 1 mg/L of the following vitamins and amino acids singly and in combination: alanine, arginine, aspartic acid, biotin, choline, cysteine, folic acid, glutamic acid, glycine, histidine, isoleucine, leucine, lysine, methionine, nicotinic acid, ornithine, phenylalanine, proline, pyridoxine, riboflavin, serine, thiamine, tryptophan, tyrosine and valine (Vekton Co., St.-Petersburg, Russia). The strains that could restore growth on supplemented CZ and form colonies similar to that on PSA were confirmed as auxotrophic for the relevant component.

In additional tests, the strains were cultivated on CZ supplemented with 0.0001, 0.001, 0.1, 1 and 10 mg/L biotin and 0.001, 0.1, 1, 10 and 100 mg/L thiamine.

### 2.7. Statistical Analysis

All experiments were repeated at least twice (total *n* ≥ 4) and found to be in agreement. The data were subjected to non-parametric ANOVA (STATISTICA 10.0), and the statistically significant difference was *p* < 0.05.

## 3. Results

### 3.1. PCR Amplification

The morphological identification of all 49 *F. langsethiae* strains was confirmed by PCR reactions with the species-specific primer pair PfusF/FlanR. DNA of all strains used in the study had clear positive reactions with the primers and formed an expected PCR product size of 300 bp [3]. This procedure makes it possible to draw reliable conclusions about *F. langsethiae* identification and allows the discriminate the *F. langsethiae* from closely related and morphologically similar species, such as *F. sporotrichioides* and *F. sibiricum*. In addition, *F. langsethiae* strains were divided into two subgroups based on the length of the IGS product amplified with the primers CNL12/PulvIGS. The strains producing amplicons of the expected size of 750 bp belonged to subgroup I, whereas the strains in subgroup II produced amplicons of 610 bp.

**Figure 1:**
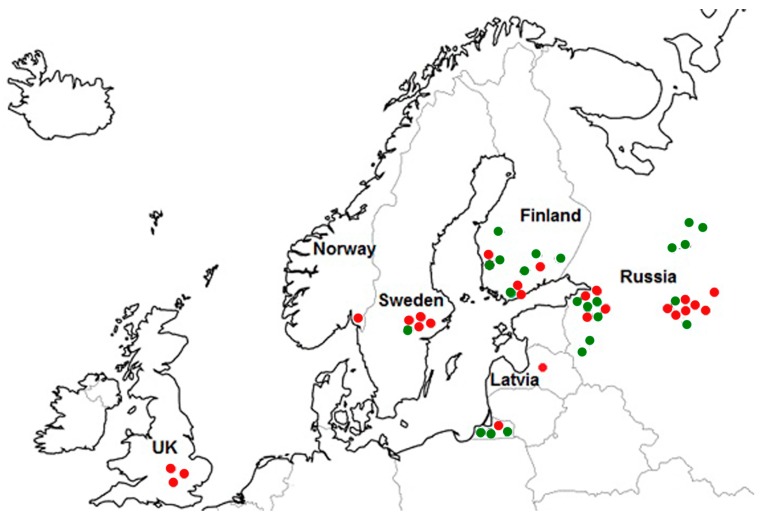
The origin of *F. langsethiae* strains belonging to different subgroups: ● (green dots)—subgroup I; ● (red dots)—subgroup II.

The geographic distributions of both *F. langsethiae* subgroups are marked on the map (Figure 1). Among 28 strains from Russia, 15 belong to subgroup I and 13 to subgroup II. Of the 11 strains from Finland, seven belong to subgroup I and four belong to subgroup II. One strain of subgroup I and four strains in subgroup II were detected among the strains from Sweden. Our findings pertaining to the strains from Sweden are consistent with previous results [31]. One strain from Latvia was assigned to subgroup II. All of the strains from the UK (three) and Norway (one) belonged to subgroup II.

### 3.2. Morphological Studies 

**Figure 2:**
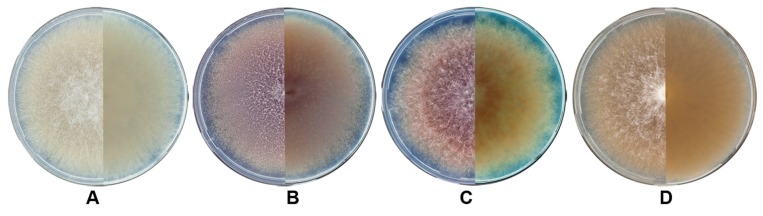
The colony phenotypes of *F. langsethiae* from above (the left part) and in reverse (the right part) on potato-sucrose agar (PSA) after incubation at 24 °C for seven days in darkness: (**A**) colorless, (**B**) violet, (**C**) pale red, (**D**) peach.

*F. langsethiae* colonies on PSA exhibited mycelium with a typically powdery appearance from above. In total, the colony of *F. langsethiae* on PSA could be characterized by four colours (colourless, peach, a shade of violet and pale red) (Figure 2). Among the analysed strains originating from the northern territory, we detected three distinct phenotypes: colourless, violet and pale red. The peach phenotype has been found only among *F. langsethiae* strains originating from the southern areas of the habitat (in preparation).

The reversal of cultures is reflected by the colour of the upper mycelial mass with the same hues but with varying intensity. The strains on CZ typically exhibited less pigment compared with PSA. The effects of glucose or sucrose which were used in CZ as carbon sources on growth and morphology of *F. langsethiae* have not been detected.

The key characteristic of the studied strains was stable in culture. Colourless and violet colonies among the strains in subgroup I were detected in equal proportions. For the strains in subgroup II, the pale red colour was the more common phenotype (50%). Thirty-one percent of the strains were colourless and 19% had violet hues.

### 3.3. The Growth Rate of Strains at Different Temperatures and Media

**Figure 3:**
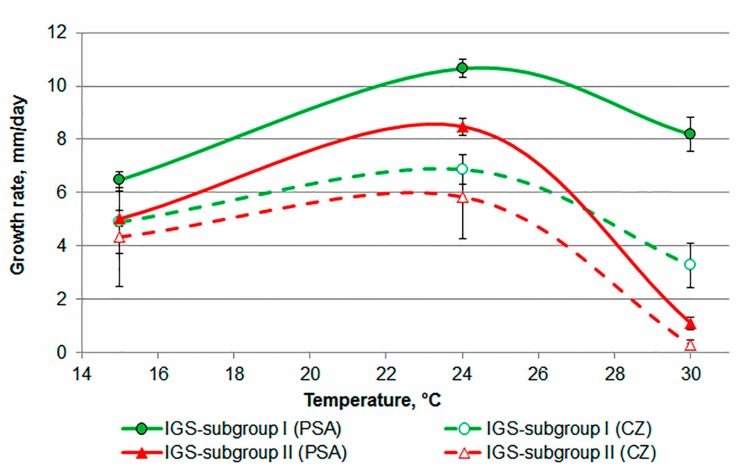
The growth curves of the *F. langsethiae* strains belonging to the subgroups I and II on PSA and CZ at the different temperatures.

The optimum growth temperature for all 49 *F. langsethiae* strains on agar media was 24 °C. The speed of growth was on average 9.5 ± 0.2 mm/day on PSA and 6.2 ± 0.7 mm/day on CZ (Figure 3). Multivariate analysis demonstrated significant effects of the subgroups, temperatures and media on the growth rate of all strains (*p* < 0.0001) and their interaction (*p* < 0.01).

On average, the size of colonies from strains in both subgroups cultivated on PSA was increased compared with CZ at all of the tested temperatures. On average, statistical differences in the growth rates at 24 and 15 °C were detected for the subgroups on the used media. On PSA, the average growth rates of strains in subgroup I and II were 6.5 ± 0.1 and 5.0 ± 0.1 mm/day at 15 °C respectively. At the optimum temperature of 24 °C, these differences were more clear: 10.6 ± 0.1 mm/day for strains in subgroup I and 8.5 ± 0.1 mm/day for strains in subgroup II.

All of the *F. langsethiae* strains in subgroup I demonstrated normal growth at 30 °C on PSA: 8.2 ± 0.3 mm/day. On CZ at 24 and 30 °C, most of the strains in subgroup I formed typical *F. langsethiae* colonies, whereas four strains originating from the Arkhangelsk region of Russia (MFG 225401, MFG 225402, MFG 225405, MFG 225406) were heavily inhibited and formed a snowflake colony.

The subgroup II strains could not grow at 30 °C on both the used media. Moreover, most of the strains in subgroup II barely showed growth on CZ at 24 °C: 3.2 ± 0.3 mm/day on average. However, two strains from subgroup II (JF-2015/32 from Sweden and MFG 220101 from Russia) did not exhibit the common features of subgroup II. These strains formed the typical for *F. langsethiae* colonies on PSA and CZ at 30 °C, as well as on CZ at 24 °C.

It should be emphasized that the abundance of aerial mycelium produced by the strains belonging to both subgroups varied more greatly than the diameter of the colonies. In contrast to most of the strains in subgroup I, which formed a relative profusion of a visible mass of filaments on CZ, most of the strains in subgroup II formed weakly visible filaments on this medium. Their colonies resembled snowflakes but occasionally exhibited a wide spread on the surface of agar in the Petri dish. These outspread hyphae consisted of swollen and malformed cells that differed from normal cells in the thickness of the cell wall and the typical abundant microconidia formation for *F. langsethiae* was not detected.

Therefore, quantitative parameters, such as the growth rate, are better estimated in liquid media by measuring the fungal biomass weight than growth on agarized media by measuring the colony diameter. The dry weight of the biomass of strains belonging to subgroup I and cultivated on liquid CZ at 24 °C was 0.6 ± 0.1 g on average. For the strains in subgroup II, this parameter was significantly less (0.04 ± 0.02 g on average). Obviously, the strains in subgroup II require some components for maintenance, growth and reproduction.

### 3.4. The Toxin Production Ability of Strains Grown on Different Media

The production of mycotoxins by the *F. langsethiae* strains belonging to subgroups I and II grown on PSA was similar for T-2 toxin (29.8 ± 3.2 and 45.2 ± 3.9 ppm on average) and DAS (1.4 ± 0. 2 and 2.3 ± 0.5 ppm on average).

Media cultivation significantly affected the mycotoxin production of the *F. langsethiae* strains (*p* < 0.015). Most of the strains in subgroup I on CZ produced large amounts of T-2 toxin (48.71 ± 5.78 ppm on average) and DAS (3.13 ± 0.88 ppm on average). Four strains (MFG 225401, MFG 225402, MFG 225405 and MFG 225406), however, produced T-2 toxin levels near the detection limit of the method (0.05–0.07 ppm) and did not produce DAS.

Under the same conditions, most of the strains belonging to subgroup II were not able to produce mycotoxins. However, two strains (JF-2015/32 and MFG 220101), in comparison with other strains in subgroup II, atypically produced large amounts of T-2 toxin (63.1 and 73.0 ppm) and DAS (1.4 and 0.3 ppm) on CZ.

### 3.5. Identification of Auxotrophic Mutants

**Figure 4:**
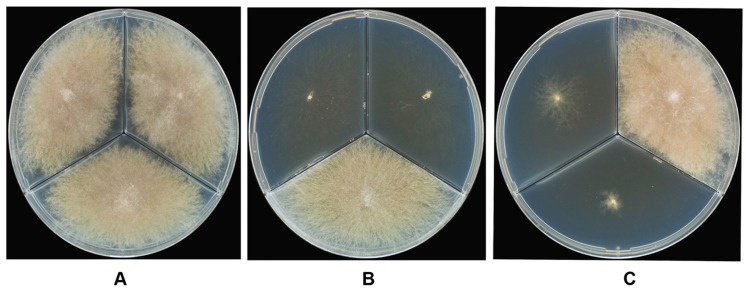
The growth behavior of *F. langsethiae* strains on CZ with the corresponding nutrients added in three sections of Petri dish: the left—vitamin-free CZ; the right—CZ + 0.01 mg/L biotin; the bottom—CZ + 0.1 mg/L thiamin. (**A**) The prototroph MFG 220101; (**B**) The thiamine auxotroph MFG 225405; (**C**) The biotin auxotroph MFG 11110. Their growth states were recorded and pictures were taken after cultivation at 24 °C for 10 days in darkness.

The above results led to the working hypothesis that the strains that were unable to grow completely on vitamin-free CZ were natural auxotrophic mutants that required certain nutrient for growth and needed to obtain those compounds from the medium. In total, 28 hypothetical auxotrophic mutants (four in subgroup I and 24 strains in subgroup II) were screened for growth stimulation by 25 vitamins and amino acids. The obligate requirement of thiamine for growth suggested that four strains in subgroup I were auxotrophic for thiamine (MFG 225401, MFG 225402, MFG 225405 and MFG 225406). The strains in subgroup II that grew on CZ supplemented with biotin, which exhibited colony phenotypes comparable with the prototrophic strains, were considered to be auxotrophic mutants for biotin (Figure 4). In addition to CZ, the other components singly or in combination did not stimulate growth.

When auxotrophic strains were grown on CZ for routine tests, the concentrations of compounds required for nutritional complementation were not critical, given the low amounts of vitamins that stimulated the growth and formation of more branched and abundant mycelia compared with growth on medium lacking vitamins.

The results reaffirmed that increasing the biotin concentration in CZ did not result in significant differences in the growth rate of the auxotrophic strains in subgroup II at 7.7 ± 0.6 mm/day (this value corresponds to growth on PSA at 24 °C). A minimal concentration of thiamine (0.01 mg/L) promoted the growth of four auxotrophic strains in subgroup I to 9.0 ± 0.5 mm/day.

## 4. Discussion

Our investigations revealed that 24 of 49 *F. langsethiae* strains were spontaneous auxotrophs in biotin and four of the strains required thiamine. Prototrophic strains that synthesize all essential biochemical compounds for growth can form typical *F. langsethiae* colonies on all used media. The auxotrophic strains do not grow on minimal media due to their inability to synthesize specific compounds.

In our laboratory, we commonly use handmade PSA to isolate fungi from plant substrates for routine tests or strain maintenance, and on this media, we did not notice the significant differences in growth between *F. langsethiae* strains. We were first intrigued by the lack of growth of some *F. langsethiae* strains from the northern area of Europe on synthetic medium compared with groups of fungi isolated from different central and southern regions of the European part of Russia [32]. Synthetic media are, generally, a good source of nitrogen, but they are vitamin-free. In contrast, PSA is composed of fresh potato and also contains carbon, nitrogen and vitamins [33]. 

Biotin (vitamin B7, vitamin H) and thiamine (vitamin B1) are water-soluble vitamins that play an essential role as a cofactor in enzyme-catalysed carboxylation reactions. In addition to its role as a cofactor, biotin has multiple roles in gene regulation. Thiamine (thiamine pyrophosphate) is a cofactor of a number of important enzymes in carbohydrate and amino acid metabolisms. A compilation of publications reveals the widespread nature of both biotin and thiamine auxotrophy within Ascomycota [33,34], including the *Fusarium* fungi [35]. 

Auxotrophs require vitamins because they have lost one or more of the essential genes involved in vitamin biosynthesis [36,37,38]. It is clear that much further work will be needed on biotin and thiamine biosynthesis pathways in *F. langsethiae*.

Prototrophic and auxotrophic strains of *F. langsethiae* could not have been phenotypically identified on basis of their growth on rich organic media. On PSA, natural auxotrophic mutants showed growth rates similar to those of phototrophic strains and eventually form the same colony phenotype. The *F. langsethiae* strains cultivated on PSA exhibited colourless, pale red and violet colony types. It was noticed that colour of colony was much more intensive on PDA in compare with PSA and especially with CZ, if the strain is able to produce any pigment. The differences in pigmentation of *F. langsethiae* strains grown on the media have also been detected and minutely described in other scientific publications [1,10,39,40]. 

The secondary metabolite aurofusarin is responsible for the production of the red/yellow pigment [41]. Aurofusarin was detected in trace amounts in five of 23 *F. langsethiae* strains [13]. In certain *F. langsethiae* strains (FL201059, IBT 9951, BBA 70945, ITEM 3602, and NRRL 54940), however, aurofusarin and the precursor rubrofusarin were not detected and the corresponding gene cluster was lacking in the fragmented draft genome [42]. We can therefore assume that the *F. langsethiae* strain isolated from Norwegian oats and chosen for genome sequencing had the colourless colony phenotype.

Among our collection of *F. langsethiae* strains originating from north Europe, the strains of both subgroups were detected in equal proportions. The overwhelming majority of the strains belonging to subgroup II (92.3%) were spontaneous auxotrophic mutants, which obligatorily required biotin for growth. Most of the strains belonging to subgroup I (83%) were prototrophs. These different features were clearly supported by the measures of growth of the strains on CZ agar and particularly by the biomass weight after cultivation in CZ liquid medium.

In each subgroup, a few strains were identified that did not need the specific nutrients typically required for most of the other strains of the subgroup (Table 2). Four *F. langsethiae* thiamine-dependent auxotrophic strains of subgroup I acted similarly to the biotin auxotrophic strains of subgroup II, except that they were able to grow at 30 °C on PSA. Oddly enough, all four strains auxotrophic for thiamine were isolated from oat grains cultivated from one location in the Arkhangelsk region of Russia. This territory is the most northern boundary area in Russia where *F*. *langsethiae* is detected.

Among the strains in subgroup II, two strains did not follow the general trend of a biotin requirement as the ingredient that promotes growth. These two strains were atypical for subgroup II and grew on CZ as they were able to synthesize all essential biochemical compounds for their growth. One strain was detected among 10 *F. langsethiae* strains originating from the Vologda region of Russia, and one strain was identified among five strains from Sweden.

The partial IGS sequences of (520–2237 bp) of seven strains in subgroup I and three strains in subgroup II, which were tested in this study, were previously obtained and deposited in the NCBI GenBank database [5]. The main difference between subgroup II and subgroup I is based on the deletion of a 140 bp amplicon in the IGS region that is amplified by the CNL12/PulvIGSr primer pair and can be used to clearly differentiate strains belonging to one or another subgroup [3,4,5]. We showed that this deletion is linked with the heat sensitivity of *F.* *langsethiae* strains in subgroup II, which failed to grow at 30 °C on PSA. In previous study, the *F. langsethiae* strains belonging to subgroup I originated mainly from Norway or Denmark, while those from subgroup II were detected among strains from Austria and the Czech Republic [3]. In our cultural collection of *F. langsethiae* (184 strains), representatives of subgroup II were detected only among the strains that originated from northern territories (data not shown). It was previously observed that the growth rates of strains from the north-western region were significantly reduced at 30 °С compared with strains from the central and the southern regions of Russia [32]. Apparently, the high temperatures that are typical of southern regions during the growing season eliminate the existence of *F. langsethiae* subgroup II. The natural conditions of northern territories might provide nutrients and allow particular auxotrophic mutations to escape purifying selection by high temperatures. This is an obvious reason as to why the auxotrophic *F. langsethiae* strains that represent the vast majority of subgroup II are found in cereals only in the northern borders of the area. In the absence of other suppression factors, auxotrophic strains can drift to high frequency. The ratio of prototrophic and auxotrophic *F. langsethiae* strains obtained from the northern European part of Russia was 15 to 13. Among 11 strains from Finland, the proportion of prototrophs and auxotrophs was seven to four. Five strains from Sweden were classified as two prototrophic and three auxotrophic *F. langsethiae* strains respectively. All three analysed strains from the UK and one strain each from Latvia and Norway were auxotrophic for biotin.

In experiments performed by Italian researchers [19], isolates were cultivated on PDA at three temperatures (15, 25 and 30 °C). Significant differences in the average mycelial growth rate per day were noted among the 24 examined isolates. The average optimum growth temperature for all of the Italian isolates was 25 °C, with lower growth rates at 15 °C and especially at 30 °C (2.2–5.7 mm/day). We hypothesized that the isolates that exhibited a reduced growth rate at 30 °C belonged to subgroup II and could be biotin-auxotrophic mutants because these *F. langsethiae* strains were isolated from rich substrates, such as grain placed on wet filter paper.

In this paper, we suggest a simple method for identifying the auxotrophic mutants of *F. langsethiae*. The technique is based on the differential heat sensitivity of strains grown on PSA at temperatures of approximately 30 °C and the parallel cultivation of strains on vitamin-free synthetic media (such as CZ) at an optimal growth temperature of 24 °C. If these strains grow on PSA at 30 °C and not on vitamin-free media at 24 °C, they are considered to be thiamine-dependent auxotrophic strains of subgroup I. If the strains failed to grow both on PSA at 30 °C and CZ at 24 °C, they are biotin-dependent auxotrophic strains of subgroup II. Previously, we also tested the strains on PDA (Scharlau, Barcelona, Spain) and statistical differences in the growth rates of the strains cultivated on PDA and CZ were not detected.

In cases where *F. langsethiae* strains are isolated from plant material on a synthetic medium, all phenotype growth due to the necessary nutrients present in the plant substrates. Sub-culturing on pure culture media that lack vitamins can lead to a high probability of loss of auxotrophic strains. In instructions for the preparation of culture media is mentioned the importance of enrichment of pure media with a solution of vitamins that make them suitable for growth of diversity microorganisms [31] and we are clearly confronted with the importance of this step. Successful cultivation of *F. langsethiae* can be achieved by the use of rich media that stimulates the growth all phenotypes without the restriction of auxotrophic strains. Under these growth conditions, any failure of the fungal growth significantly changes our knowledge about the composition of microorganisms. This phenomenon is important especially when the studies are linked with identification of toxin-producing fungi.

*F. langsethiae* is frequently detected in grains when using species–specific primers; quantitative PCR offers an efficient estimation of the biomass of individual species. Generally, a good correspondence was noted between DNA of *F. langsethiae* and the T-2/HT-2 toxins detected in grain samples [43,44,45,46,47].

However, *F. langsethiae* is more frequently detected in grain samples by PCR compared with the microbiological method of isolation [1,48]. According to Edwards et al. [49], this finding could result from a large number of seeds that have *F. langsethiae* on the surface, which is killed during surface sterilization. In addition, this species grows slowly and was potentially overgrown or suppressed by other faster-growing species. Our additional explanation suggests that the isolation of auxotrophic strains is complicated because *F. langsethiae* normally thrives within host tissues, which have a rich set of nutrient elements that are difficult to replicate on vitamin-free media.

The requirement for biotin and thiamine by *F. langsethiae* indicates that these vitamins are available in the plant tissue from which this species was isolated. Biotin is wildly distributed in plants, particularly in whole grains. In oat kernels, biotin is present at 0.19 mg/kg, which is a 10.0-fold increase compared with wheat kernels [50,51]. According to Koehler et al. [52], thiamine is present in oats at 6.7 mg/kg (average values), which is a 1.5- to 1.9-fold increase compared with other cereal plants. A large amount of these vitamins in oats possibly explains why oats are a preferred crop for *F. langsethiae*. This fungus and type-A trichothecenes are frequently found in these vegetated plants and harvested kernels [53,54]. In our research, the relation between the occurrence of auxotrophs and the plant source was not established.

The genetic changes involved in basic metabolism may give rise to changes in plant and fungi interactions. In the future, it will be interesting to compare the pathogenicity of auxotrophic and prototrophic strains. However, these differences are unlikely to be significant in the presence of even small amounts of biotin in the plant tissue. Moreover, the study of change in pathogenicity of auxotrophic strains is problematic due to complexity inoculation of plants by *F. langsethiae*.

It was previously shown that basal nutrient elements in suitable substrates, such as carbohydrates and certain amino acids, are favourable for trichothecene toxin production in vitro [55,56,57,58,59]. Efficient toxin production is typically observed on cereal-based substrates [58,60]. Both cereal flour and rice flour media promoted toxin production [58]. These media contain yeast extract at 1 g/L, which is rich in biotin [61]. In our experiments, phototrophic strains are able to produce comparable levels of mycotoxins on the media regardless of the presence of vitamins. In media without essential vitamins, a significant reduction in the growth of auxotrophic strains leads to a fall in T-2 toxin and DAS formation. The production of mycotoxins is not affected by auxotrophic mutations on nutritionally complete media, and the amounts are similar for prototrophic *F. langsethiae* strains.

We are not aware of previous data pertaining to the isolation of auxotrophic mutants in *F. langsethiae*. The first obvious future step would be to elucidate the causal mechanism of the change in biotin and thiamine requirements. These results highlight the need for subsequent investigations into genes essential for biotin/thiamine biosynthesis.

Auxotrophy for a specific nutrient in *F. langsethiae* can be a useful marker for revealing any interaction with plants. Further research is needed to unravel the biological significance of auxotrophy of *F. langsethiae* and to understand the broad aspects of its physiology. The revealed auxotrophy may shed light on the, till now, poorly understood nature of plant-fungus interaction, reproduction, dissemination and survival of *F. langsethiae*.

## Figures and Tables

**Table 1:** *Fusarium langsethiae* strains used in the present work.

No.	VIZR Collection No.	No. in Other Collections/GenBank Accession No.	Year of Isolation	Geographic Origin (Country, Region)	Source	Growth on Basal Czapek (CZ) **	Intergenic spacer (IGS)-Subgroup
1 *	MFG 203401	—	2013	Russia, Leningrad	oat	+	I
2 *	MFG 11103	—	2014	Russia, Leningrad	triticale	−	II
3 *	MFG 217011	—	2014	Russia, Leningrad	oat	+	I
4 *	MFG 217012	—	2014	Russia, Leningrad	oat	+	I
5 *	MFG 217701	—	2014	Russia, Leningrad	oat	+	I
6 *	MFG 217702	—	2014	Russia, Leningrad	oat	−	II
7	MFG 133601	—	2010	Russia, Leningrad	oat	−	II
8	MFG 93001	—	2008	Russia, Leningrad	barley	−	II
9	MFG 11020	NRRL 53437/HM060290	2008	Russia, Pskov	oat	+	I
10	MFG 217903	—	2014	Russia, Pskov	oat	+	I
11 *	MFG 220102	—	2014	Russia, Vologda	oat	+	I
12 *	MFG 223301	—	2014	Russia, Vologda	oat	−	II
13 *	MFG 223302	—	2014	Russia, Vologda	oat	−	II
14	MFG 223401	—	2014	Russia, Vologda	oat	+	I
15 *	MFG 223402	—	2014	Russia, Vologda	oat	−	II
16	MFG 224306	—	2014	Russia, Vologda	oat	−	II
17	MFG 220101	—	2014	Russia, Vologda	oat	+	II
18	MFG 100601	—	2008	Russia, Vologda	oat	−	II
19	MFG 100602	—	2008	Russia, Vologda	oat	−	II
20	MFG 223407	—	2014	Russia, Vologda	oat	−	II
21	MFG 225401	—	2014	Russia, Arkhangelsk	oat	−	I
22	MFG 225402	—	2014	Russia, Arkhangelsk	oat	−	I
23	MFG 225405	—	2014	Russia, Arkhangelsk	oat	−	I
24	MFG 225406	—	2014	Russia, Arkhangelsk	oat	−	I
25	MFG 103506	—	2008	Russia, Kaliningrad	oat	+	I
26	MFG 103507	—	2008	Russia, Kaliningrad	oat	+	I
27	MFG 11021	NRRL 53538	2008	Russia, Kaliningrad	oat	+	I
28	MFG 55201	—	2005	Russia, Kaliningrad	oat	−	II
29 *	—	FI 2004/57	2004	The UK	oat	−	II
30 *	MFG 11037	FI 062/1	—	The UK	oat	−	II
31 *	—	FI 026/1	—	The UK	oat	−	II
32	—	9822–219–1F	—	Norway, Østfold	oat	−	II
33	—	54–Fin 03	2003	Finland	—	−	II
34	—	52–Fin 03	2003	Finland	—	+	I
35 *	MFG 11027	NRRL 53409/HM060272	2003	Finland, Etelä-Karjala	barley	+	I
36	MFG 11028	NRRL 53419/HM060288	2003	Finland, Satakunta	oat	+	I
37	MFG 11030	NRRL 53411/HM060282	2003	Finland, Satakunta	oat	+	I
38	MFG 11029	NRRL 53410/HM060286	2003	Finland, Satakunta	oat	−	II
39 *	MFG 11033	NRRL 53414/HM060285	2003	Finland, Häme	wheat	+	I
40 *	MFG 11032	NRRL 53413/HM060284	2003	Finland, Uusimaa	wheat	+	II
41 *	MFG 11031	NRRL 53412/HM060283	2003	Finland, Uusimaa	wheat	−	II
42	MFG 11034	NRRL 53417/HM060287	2003	Finland, Uusimaa	oat	+	I
43 *	MFG 11035	NRRL 53418/HM060273	2003	Finland, Etelä-Pohjanmaa	wheat	+	I
44	MFG 11110	JF-2015/01	2015	Sweden	oat	−	II
45	MFG 11111	JF-2015/14	2015	Sweden	oat	−	II
46	MFG 11112	JF-2015/02	2015	Sweden	oat	+	I
47	MFG 11113	JF-2015/30	2015	Sweden	oat	−	II
48	MFG 11114	JF-2015/32	2015	Sweden	oat	+	II
49	MFG 232405	—	2016	Latvia	oat	−	II

* The strains were additionally cultivated in liquid CZ; ** “−“ lack or barely of growth, “+” typical growth for *F. langsethiae*.

**Table 2:** Characterization of *F. langsethiae* strains belonging to the different subgroups.

Subgroup	No. of Strains	Growth on PSA at 30 °C	Growth on CZ at 24 °C	Mycotoxins Production on CZ	Phenotype/Nutritional Requirement
I	19	+	+	high	prototroph
	4 *	+	−	low	auxotroph for thiamin
II	24	−	−	low	auxotroph for biotin
	2 **	−	+	high	prototroph

“−“ Lack or barely of growth, “+” typical growth for *F. langsethiae*; * MFG 225401, MFG 225402, MFG 225405, MFG 225406; ** JF-2015/32, MFG 220101.
